# Clinical Validation of Eleven Formulas for Calculating LDL-C in Iran

**DOI:** 10.30699/ijp.2020.110379.2174

**Published:** 2020-07-18

**Authors:** Fereshteh Atabi, Reza Mohammadi

**Affiliations:** 1 *Department of Biochemistry and Biophysics, Faculty of Advanced Sciences and Technology, Tehran Medical Sciences, Islamic Azad University, Tehran, Iran*; 2 *Department of Biochemistry, Faculty of Medicine, Tehran Medical Sciences, Islamic Azad University, Tehran, Iran*

**Keywords:** Cardiovascular disease, Friedewald formula, HDL, LDL, TG

## Abstract

**Background & Objective:**

Concentration of low-density lipoprotein (LDL) is a known risk factor for cardiovascular disease which is routinely measured or calculated as LDL-C in clinical laboratories. In order to decrease the cost, instead of its measuring, it is recommended to calculate it using multiple formulas that have been introduced up to now. The aim of this study was to assess the results of various formulas and comparison of these results with those of measuring method and to clarify the best formula for the Iranian population.

**Methods:**

Concentrations of total cholesterol (TC), triglyceride (TG), cholesterol of high-density lipoprotein (HDL-C) and LDL-C in serums of 471 overnight fasting individuals were measured and also LDL-Cs of these samples were calculated by eleven different formulas according to their TC, TG, and HDL-C concentrations. Subsequently, results of measured and calculated LDL-C were analyzed statistically by paired t-test, correlation coefficient, and Passing-Bablok regression. In addition, for clinical evaluation, the differences between calculated and measured mean results were calculated and compared with an allowable total error.

**Results:**

Paired t-test unraveled a significant difference between the results of measured and calculated LDL-C by various formulas. But for some formulas, these differences were not clinically significant. The best clinical and statistical agreement (correlation coefficient) was obtained by the Friedewald equation.

**Conclusion:**

By using validated methods which have correct calibration and control system for measuring TC, TG, and HDL-C, we can use the Friedewald formula for calculating LDL-C in serum samples with TG up to 400 mg/dL.

## Introduction

Cardiovascular disease is the most common cause of morbidity and mortality worldwide (1). Increased plasma low density lipoprotein (LDL) concentration is an important known risk factor for this disease (2-4). So, one of the main goals in preventing cardiovascular disease is to reduce plasma or serum LDL concentration (1,3). Ultracentrifugation is the reference method for measuring serum LDL concentration and LDL subfraction distribution which is cumbersome and has a high cost (4,5). Therefore, Ultracentrifugation is not common in routine clinical laboratories (1). Some clinical laboratories measure cholesterol component of LDL (LDL-C) as an estimate of LDL concentration. These homogenous direct LDL-C methods rely on measuring cholesterol of LDL particles in the presence of other lipoprotein particles which have been prevented from participating in measuring cholesterol reaction. In the third method, LDL-C is calculated according to the total cholesterol (TC), triglyceride (TG), cholesterol of high density lipoprotein (HDL-C) and LDL-C and applying Friedewald equation (6).

Although LDL-C homogenous direct methods are precise and can be used in autoanalyzer instruments, in some cases, such as presence of abnormal lipoproteins, they have differences in ultracentrifugation methods. When triglyceride concentration is lower than 400 mg/dL, using direct method has no advantages to the calculation method. On the other hand, calculation method is associated with decreasing the cost. National Cholesterol Education Program- Adult treatment panel III (NCEP-ATP III) recommends to use calculated LDL-C, instead of LDL-C direct methods, for serum samples with TG concentration up to 400 mg/dL. According to this recommendation, running of direct LDL-C would only enhance the expense (3,7). Friedewald equation is based on four assumptions: 1) In 12 hours overnight fasting state, chylomicron is not presented in circulation and total plasma cholesterol concentration is primarily carried in VLDL, LDL, and HDL forms; 2) Essentially all plasma TG are carried by VLDL; 3) VLDL-TG /cholesterol ratio is constant; and 4) Cholesterol concentration of VLDL (VLDL-C) is one fifth of TG concentration (7). According to these assumptions, Friedewald equation is as LDL-C = Tc – (HDL-C + TG/5) (8). In this equation, units of all analysts are according to mg/dL.

In spite of extensive application of Friedewald equation for calculating LDL-C concentration of serum samples with TG up to 400 mg/dL, several studies have shown that this equation lack good performance in various conditions (1,2,8-14). Thereby, multiple groups are continuously evaluating the Friedewald equation accuracy in different population and diseases (1,3). According to these studies, more than ten equations were introduced for calculating LDL-C. 

The aim of this study was to compare results of calculating LDL-C by eleven introduced equations, including Friedewald equation, with results of direct LDL-C measuring method and determining the best equation for Iranian population. In this study, we also compared the results of statistical analysis and clinical requirements which are necessary for validating methods in clinical laboratories.

##  Materials and Methods

**Grouping**

The accuracy of LDL-C calculation is affected by TG concentration. Errors in LDL-C calculation become noticeable in triglyceride concentrations over 200 mg/dL and become unacceptably large at triglyceride concentrations over 400 mg/dL. On the other hand, as Friedewald equation is valid only for samples with TG concentrations up to 400 mg/d, 29 samples with TG concentrations more than 400 mg/dL were excluded and 471 remained samples were classified in four groups with TG concentrations up to 100 mg/dL, 101 to 200 mg/dL, 201 to 300 mg/dL, and 301 to 400 mg/dL, which their sample numbers were 185, 204, 70, and 12, respectively. 

**Study Population**

Study population included 500 staffs of Ava Protein Company, a meat and poultry Products Company in Tehran, Iran, which during November 2018 participated in annual health screening. Venous blood samples of these individuals were collected in redtop vacuum tubes containing coagulation accelerator and gel separator after 12 to 14 hours overnight fasting (5). Serums were separated within 2 hours by centrifugation of coagulated whole bloods at 500 g for 10 minutes. Then separated serums were transported into disposable tubes and refrigerated.

**Lipid Profile Analyses**

Lipid profile, including TC, TG, HDL-C, and LDL-C, were analyzed daily within 6 hours after blood collection. We used Pars Azmoon kits for lipid analyses, which are the common biochemical kits in Iran. For measuring serum total cholesterol by Pars Azmoon kit, cholesteryl esters are hydrolyzed by cholesteryl esterase and then 3-OH group of cholesterol is oxidized and finally hydrogen peroxide produced by this reaction is quantified by producing colored product during peroxidase reaction. Principle of measuring of serum triglyceride by Pars Azmoon kit is as principle of cholesterol measuring, except that glycerol is produced by action of lipase on triglyceride and hydrogen peroxide is produced during glycerol oxidase reaction. For measuring serum LDL-C and HDL-C, Pars Azmoon kits use direct methods in which blocking agents inhibit lipoproteins other than LDL-C and HDL-C to participate in cholesterol measurement, respectively. 

Pars Azmoon kits were installed on Roche Hitachi 912 Chemistry Analyzer and calibrated by calibrator recommended by kit producer. For calibration assurance, calibration verification was accomplished daily. Also for assurance of accurate performance of the methods, two level quality control (QC) materials, including normal and high concentration levels, were used in each run and the results of QC materials were interpreted according to sigma metrics.

**LDL-C Calculation**

In order to calculate LDL-C, we used eleven equations which are listed in Table 1. 

**Table 1:** Formulas which were used to calculate LDL-C

**Formula**	**Equation**	**Reference**
**Friedewald**	LDL-C = TC – HDL-C – (TG/5)	6, 9, 10
**Puavilai**	LDL-C = TC – HDL-C – (TG/6)	
**Vujovic**	LDL-C = TC – HDL-C – (TG/6.85)	
**Hattori**	LDL-C = (0.94 × TC) – (0.94 × HDL-C) – (0.19 × TG)	6, 9, 10
**Anandaraja**	LDL-C = (0.9 × TC) – (0. 9 × [TG/5]) – 28	6, 9, 10
**Chen**	LDL-C = (0.9 × TC) – (0.9 × HDL-C) – (0.1 × TG)	6, 10
**Cordova**	LDL-C = 0.7516 × (TC – HDL-C)	
**Teerakanchana**	LDL-C = (0.91 × TC) – (0.634 × HDL-C) – (0.111 × TG) – 6.755	6, 9
**Ahmadi**	LDL-C = (TC/1.19) – (HDL-C/1.1) – (TG/1.9) - 38	6, 9
**DeLong**	LDL-C = TC – HDL-C – (0.16 × TG)	
**Rao**	LDL-C = [(4.7 × TC) – (4.364 × HDL-C) –TG]/4.487	

***Abbreviations:*** HDL-C, High density lipoprotein cholesterol; LDL-C, Low density lipoprotein cholesterol; TC, Total cholesterol; TG, Triglyceride.

**Data Analyses**

Data were analyzed both statistically and clinically. For statistical analyses, we used Paired t-test, correlation coefficient, and Passing-Bablok regression. Linear regression is not valid, as both comparative methods (direct measured LDL-C) and test method (calculated LDL-C) have errors. MedCalc software was used for statistical analyses.

For clinical analyses of acceptable performance of measuring methods, we used total allowable error (TEa) of 12% which is determined by NCEP (15). This TEa is for when the test is repeated once. In this situation, one third of TEa is considered for systematic error (bias) (15), two third for random error (imprecision), and, when the t-test is repeated, imprecision is decreased by $1/\sqrt[{2}]{n}$ (16) which itself results in decreasing TEa to modify TEa (mTEa) calculated as equation 

$\mathrm{mTEa\%}=\frac{2=\sqrt[{2}]{n}}{3\times \sqrt[{2}]{n}}\times \mathrm{TEa\%}$

(1-1)

When the test is repeated or samples are 400 or more, random error decreases to 5% or lower and we can ignore this error. In this case, TEa reduces to bias (one third of 12%) which equals 4%. In this study, when we divided 471 samples in four groups, numbers of samples in groups 1 to group 4 were 185, 204, 70, and 12. So, calculated mTEa for these groups are 4.6%, 4.6%, 5.0%, and 6.3%, respectively.

## Results

Mean of results of calculating LDL-C by different formulas along with the results of statistical (paired t-test) and clinically (TEa 4% for more than 400 samples) comparison of these mean values with mean values of measured LDL-C, are summarized in Table 2. There was statistically a significant difference (*P*<0.0001) between calculated mean values and measured mean value. This difference was also clinically significant for all calculations, except Friedewald, Anandaraja, and Chen formulas.

**Table 2:** Statistic and clinical comparison of calculated LDL-C mean values of different formulas with measured LDL-C mean value (104.52 mg/dL; 95% CI from 101.52 to 107.10 mg/dL).

**Formula**	**Mean**		**Paired t-test**		**Difference**		
	Value	95% CI	Statistic	TTP	Absolute	Percent	CS
**Friedewald**	106.57	103.93 to 109.21	6.051	< 0.0001	2.05	2.0	No
**Puavilai**	110.97	108.30 to 113.64	18.568	< 0.0001	6.45	6.2	Yes
**Vujovic**	113.70	116.39 to 111.00	24.860	< 0.0001	9.18	8.8	Yes
**Hattori**	99.1	102.40 to 97.43	- 13.846	< 0.0001	-4.61	-4.4	Yes
**Anandaraja**	107.88	110.51 to 105.25	6.557	< 0.0001	3.36	3.2	No
**Chen**	106.47	103.10 to 108.94	4.844	< 0.0001	1.95	1.9	No
**Cordova**	100.25	97.96 to 102.53	- 7.006	< 0.0001	- 4.27	-4.1	Yes
**Teerakanchana**	111.85	109.33 to 114.36	19.908	< 0.0001	7.33	7.0	Yes
**Ahmadi**	118.60	116.09 to 121.12	38.060	< 0.0001	14.08	13.5	Yes
**DeLong**	111.85	109.17 to 114.52	20.757	< 0.0001	7.33	7.0	Yes
**Rao**	113.19	110.41 to 114.98	23.421	< 0.0001	8.67	8.3	Yes

***Abbreviations:*** CI, Confidence interval; CS; Clinical significance; LDL-C, Low density lipoprotein cholesterol; TTP; Two-tailed probability.

**Fig. 1:**
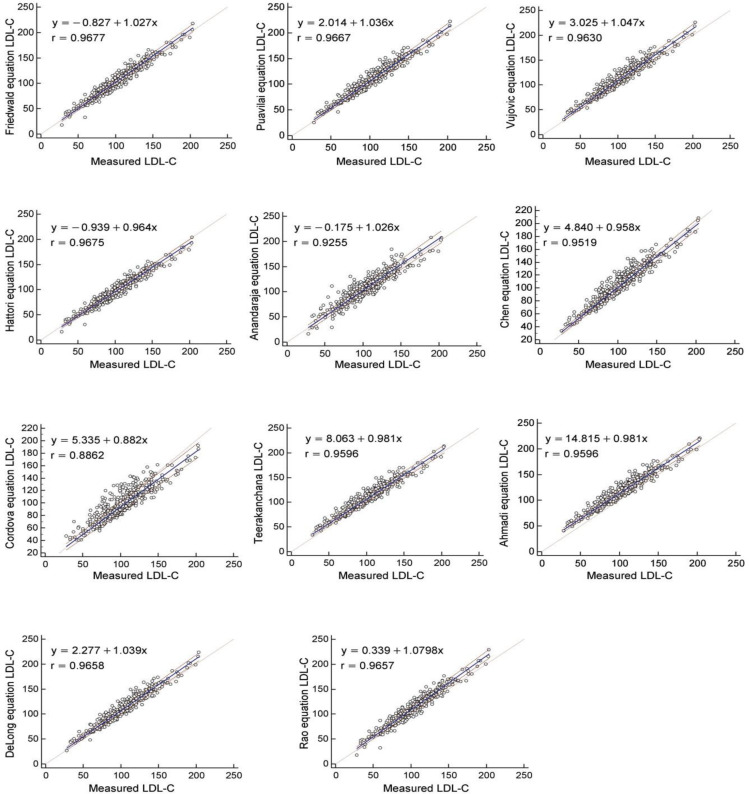
Passing-Bablok Regression analyses of calculated LDL-C values of different formulas with measured LDL-C values

Results of regression analyses of measured and calculated LDL-C values are summarized in Table 3 and shown in Figure 1. All calculated results exhibited good correlation (>0.9500) with measured results, except for Anandaraja and Cordova equations. According to Passing-Bablok regression, results of Friedewald, Puavalai, Hattori, Cordova, DeLong, and Rao equations showed constant (y intercept) systematic error and results of Chen and Vujovic showed both constant and proportional (slope), but results of Anandaraja showed none of these errors.

**Table 3:** Regression analyses of calculating LDL-C values of different formulas with measured LDL-C values

**Formula**	**Correlation coefficient**		**Passing-Bablok regression**		
	Value	95% CI	Equation	Significant difference	
				Y intercept	Slope
**Friedewald**	0.9677	0.9614 to 0.9729	y = - 0.827 + 1.027 x	No	Yes
**Puavilai**	0.9667	0.9602 to 0.9721	y = 2.014 + 1.036 x	No	Yes
**Vujovic**	0.9630	0.9558 to 0.9690	y = 3.025 + 1.047 x	Yes	Yes
**Hattori**	0.9675	0.9612 to 0.9728	y = - 0.939 + 0.964 x	No	Yes
**Anandaraja**	0.9255	0.9114 to 0.9375	y = - 0.175 + 1.026 x	No	No
**Chen**	0.9519	0.9426 to 0.9597	y = 4.840 + 0.958 x	Yes	Yes
**Cordova**	0.8862	0.8651 to 0.9041	y = 5.335 + 0.882 x	No	Yes
**Teerakanchana**	0.9596	0.9518 to 0.9662	y = 8.063 + 0.981 x	Yes	No
**Ahmadi**	0.9596	0.9518 to 0.9662	y = 14.815 + 0.981 x	Yes	No
**DeLong**	0.9658	0.9591 to 0.9714	y = 2.277 + 1.039 x	No	Yes
**Rao**	0.9657	0.9591 to 0.9713	y = 0.3369 + 1.080 x	No	Yes

***Abbreviations:*** LDL-C, Low density lipoprotein cholesterol; CI, Confidence interval.

Table 4 depicts grouping of samples according to TG concentrations along with statistical and clinical comparison of Friedewald calculated LDL-C mean values and measured mean values. Selection of Friedewald results in this analysis was due to having a better agreement with results of measured LDL-C (see Tables 2 and 3). Groups 1 and 2 showed clinically significant differences, but the differences were insignificant for groups 3 and 4. There was no statistically significant difference for all four groups. 

**Table 4:** Statistics and clinical comparison of Friedewald calculated LDL-C mean values with measured LDL-C mean values according to TG concentrations

**Group**	**TG** **(mg/dL)**	**n**	**Mean (mg/dL)**		**Paired t-test**			**Diffferences**		
			Calculated	Measured	statistic	TTP	SD_diff_	Ab	(%)	C S
**1**	Up to 100	185	97.57	100.46	- 6.666	<0.000.1	5.8984	- 2.89	- 2.9	No
**2**	101 – 200	204	111.72	113.13	- 2.730	0.0069	7.3643	- 1.41	- 1.2	No
**3**	201 – 300	70	102.44	104.74	- 1.981	0.0516	9.7414	- 2.30	- 2.2	No
**4**	301 - 400	12	101.16	99.88	0.419	0.6830	10.5640	1.28	1.3	No

***Abbreviations:*** Ab; Absolute; CS; Clinical significance; LDL-C, Low density lipoprotein cholesterol; SD_diff_, Standard deviation of differences; TG, Triglyceride; TTP; Two-tailed probability.

## Discussion

LDL-C measurement is a routine test in the clinical laboratory which is used for assessing the risk of coronary heart disease. For reducing costs, LDL-C measuring is replaced by calculating LDL-C with Friedewald equation and according to the results of measured TC, TG, and HDL-C (3). During recent years, in order to assess the validity of Friedewald equation and introducing new equations, multiple studies have been performed in different countries, including Iran, which have been raised to different results. 

In 2008, Ahmadi *et al.* showed overestimation of LDL-C by Friedewald equation for samples with low TG and high TG concentrations (6). They introduced a new equation for calculating LDL-C (6) which conferred no good agreement with measured LDL-C in their studies (1,3,17). Boshtam *et al.* performed another study in Iran in 2012. On the basis of this study, they concluded that Friedewald equation overestimate LDL-C concentrations in Iranian population and recommended to measure LDL-C directly (18).

Cordova *et al.* had a study on Brazilian population and introduced a new formula for estimation of LDL-C in which TG concentration was omitted (2). In 2016, Hichem *et al.* reported their study on North Africa (Algeria) population in order to highlight the formula that calculate LDL-C more accurately than Friedewald formula on a North Africa (Algeria) population. They used different formulas for LDL-C calculation, including Friedewald, Hattori, Puavilai, Anandaraja, Ahmadi, Vujovic, and Cordova formulas. They found out that Friedewald, Puavilai, and Vujovic formulas have the highest agreement (correlation coefficient of 0.930 to 0.934). They found out that Friedewald and Puavilai calculated mean values had statistically significant differences, but this difference was not statistically significant for the Vujovic calculated mean value. Finally, they concluded that Puavilai formula was the most suitable for North Africa population (19). 

In 2019, Karkhaneh *et al.* reported their study on eight formulas for LDL-C estimation in Iranian subjects with different metabolic healthcare status according to their Fasting blood sugar (FBS), TG, TC, HDL-C, and age. But they didn't find any formula for accurate estimation of LDL-C in all subjects and concluded that Hattori and Cordova formulas could be the best alternatives for LDL-C direct measurement in Iranian population, especially for healthy subjects. It seems that it was not necessary to divide subjects to different groups according to FBS, TC, HDL-C, and age. Because, in a clinical laboratory, TG concentration is considered as the most important factor affecting on calculating LDL-C concentration (20). 

In the Friedewald formula, VLDL-C is calculated as TG/5. In order to have a better estimation of LDL-C, in Vujovic and DeLong formulas, 5 is replaced by 6 and 6.25, respectively (1,19). In 2016, Rim *et al.* by studying on Korea population concluded that using a variable factor according to TG concentration is a better approach (1).

In our study, none of the studying formulas and factors had a better performance than Friedewald equation. As shown in Tables 2 and 3, the results of Friedewald and Chen Formulas have the least differences (1.9% and 2.0%, respectively) from measured LDL-C results and between these two formulas, correlation of Friedewald formula was better (0.9677) than Chen formula (0.9519). In 2016, Chen *et al.* reported that Friedewald formula has the best correlation (0.977) in all TG concentrations (17).

There are many other studies that had evaluated different formulas for calculating LDL-C against LDL-C direct measurement. Some studies showed Frieldwald formula as the best one (21-23), some others showed that other formulas are more accurate (24-29). In contrast, Anwar et al, recommended using direct homogeneous assay in clinical laboratories for measuring LDL-C, because there is no uniformity in performance of LDL-C estimation at different TG levels (30).

An important part of discrepancies in results of multiple studies may be due to probable systematic and random errors in measuring TC, TG, HDL-C, and LDL-C concentrations. Additionally, they are due to mode of judgement and interpretation of results of statistical analysis. In most studies, statistical analysis focused on paired t-test, correlation coefficient, and regression analysis. In addition to have a direct correlation with the differences of means, result of t-test has direct and invert relation with numbers of samples and standard deviation of differences (SD_diff_), respectively. Groups 1 and 2 in Table 3 showed significant differences between mean calculated and measured values. These differences are not due to great differences of means (22.89 and 1.41, respectively), but is due to having a higher number of samples (185 and 204, respectively) and a lower SD_diff_ (5.8984 and 7.3643, respectively). In contrast, Groups 3 and 4 that had comparable mean differences (-2.30 and -1.28, respectively), showed insignificant differences. These differences are due to having a lower number of samples (70 and 12, respectively) and a higher SD_diff_ (9.7414 and 10.5640, respectively). This showed paired t-test is not a suitable criterion for judgement of results of method comparison study in clinical laboratories. Hence, it is better to have a clinical judgement by using total allowable error (TEa) and modified TEa (mTEa). As shown in Table 3, in spite of statistical differences in groups 1 and 2, clinically these differences were not significant.

## Conclusion

On the basis of this study, if validated methods are used for measuring TC, TG, and LDL-C which has the correct calibration plan and are under correct quality control plan, Friedewald formula is the best equation for calculating LDL-C concentration of serum samples with TG concentration up to 400 mg/dL. In this situations, the differences between calculated and measured LDL-C concentrations is not clinically significant and has no effect on medical decision making.
